# Mechanical response of long-span CFST arch bridges based on the hydration heat temperature effect

**DOI:** 10.1038/s41598-024-65361-1

**Published:** 2024-06-25

**Authors:** Yuexing Wu, Qiang Wen, Meihong Dai, Xinzhong Wang, Xingxin Li, Xianliang Tan

**Affiliations:** 1 School of Civil Engineering, Hunan City University, Yiyang, 413000 China; 2 State Key Laboratory of Mountain Bridge and Tunnel Engineering, Chongqing Jiaotong University, Chongqing, 400074 China; 3 School of Civil Engineering, Chongqing Jiaotong University, Chongqing, 400074 China; 4 China State Construction Bridge Co., Ltd., Chongqing, 402260 China

**Keywords:** Concrete-filled steel tube, Hydration heat, Temperature field, Mechanical response, Engineering, Civil engineering

## Abstract

As the span of concrete-filled steel tube (CFST) arch bridges increases, the hydration heat temperature effect of concrete inside steel tube becomes more severe, which increases the safety risk during the construction process. Therefore, a numerical simulation of the mechanical response of a long-span CFST arch bridge under the influence of hydration heat was carried out. First, based on the hydration heat conduction theory, a finite element model of the transient temperature field of a CFST arch rib was established. The temperature distribution of the CFST arch rib and its variation with time were revealed, and an approximate formula for the distribution of the hydration heat temperature along the radial direction of the CFST was provided. Subsequently, the variation law of the thermal stress of a CFST during hydration heat release was investigated. Finally, based on the principle of temperature equivalence, a finite element model of the overall CFST arch rib was established to examine the effect of hydration heat on the deformation of the arch rib. The results reveal that the hydration heat temperature field of the CFST arch rib exhibits nonlinear and axisymmetric characteristics. The maximum temperature of the section and the maximum temperature difference can reach 73.5 °C and 33.2 °C, respectively. Because of the influence of the hydration heat, there is a significant stress gradient in the cross section of the arch rib. A maximum radial stress of 2.08 MPa is attained, indicating a risk of concrete cracking. Additionally, the displacement along the transverse and vertical directions of the chord tube exhibits an initial increase, followed by a decrease over time. The maximum transverse displacement of the chord tube reaches 70.6 mm, while the vertical displacement reaches 117.8 mm.

## Introduction

Concrete-filled steel tube (CFST) arch bridges have been extensively constructed over the past two decades due to their advantages of high load-bearing capacity, convenient construction, and adaptability to complex terrains^1–5^. As a composite structure, steel tubes can improve the strength and ductility of concrete. Conversely, the core concrete can also significantly prevent local buckling of the steel tube, which improves the overall bearing capacity of the CFST^6^. However, with the span of CFST arch bridges exceeding 500 m^1^ and the steel tube diameter approaching 1.5 m^7^, the temperature effect becomes increasingly apparent under the influence of hydration heat, sunshine radiation, large temperature differences and other factors^8,9^. For the construction process of CFST arch bridges, the influence of the hydration heat of mass concrete is more severe, resulting in potential issues such as voids and cracking, thereby escalating construction challenges and undermining the load-bearing capacity and durability of CFST arch bridges^10–12^. Nevertheless, these crucial issues have not been addressed because the relevant design standards^13–15^ for bridges do not consider the effect of hydration heat on the mechanical properties of a CFST^10,16^. Thus, it is imperative to conduct a comprehensive investigation on the mechanical response of large-span CFST arch bridges based on the hydration heat temperature effect.

To date, several experimental and theoretical investigations have been conducted on the hydration heat temperature of CFSTs. In early studies, Chen^17^ and Han^18^ carried out temperature field measurements of hydration heat in small-size CFST members (i.e., typically with diameters less than 1 m). All tests showed that the hydration heat temperature of the CFST increases with the release of the heat of hydration, reaching a peak before gradually decreasing over time. Additionally, a temperature gradient appears across the cross section of the arch. Subsequently, as the span of CFST arch bridges increased, high-strength microexpansive concrete was employed to enhance the bond between the steel tube and concrete in large-diameter CFST arches^19–21^. Xuan^22^, Gao^7^, Yang^23,24^ and Sun^10^ implemented experimental studies and numerical simulations on the temperature variations in large-size CFST arch cross sections (i.e., typically with diameters greater than 1 m) during the hydration heat release process and further studied the effects of the structural parameters of large-size steel tubes on the temperature field induced by hydration heat in arch bridge structures. The results indicated that the maximum temperature of a large-diameter CFST can reach 81.3 °C during the hydration process, while an extremely significant temperature gradient persists across the cross section. The aforementioned studies have identified the distribution patterns and trends of the hydration temperature field. However, a simple calculation formula for its distribution in the cross section of large-span CFST arch rids has not been proposed. Therefore, further analysis is required to build upon the existing research achievements.

Researchers have conducted further analyses on the effect of hydration heat temperature on CFST structures. For instance, Xuan^22^ found that the arch rib experienced a significant temperature gradient and temperature stress due to concrete hydration heat. The influence of thermal stress should be accounted for during the construction monitoring process. Through numerical analysis, Sun^25,26^ revealed that thermal stress rapidly increases at the beginning of concrete hydration, with a significant stress gradient along the radial direction of the arch rib cross section. After reaching a peak, the stress gradually decreases, leaving residual thermal tensile stress in the concrete. Xie^16^ investigated the thermal stress in the hydration heat release process of a CFST using a finite element model and analytical study. This researcher observed that the radial thermal stress initially displayed compressive stress during the early stages of hydration, gradually transitioning to tensile stress after reaching a peak and crossing the zero-stress condition. The circumferential stress at the centre of the concrete exhibited tensile stress during the cooling stage, whereas the stress at the concrete edge showed tensile stress during the temperature increase period. Zhou^9^ studied the temperature field of large-diameter CFST arch bridges under hydration heat and other factors, and proposed the optimization method on transverse perfusion sequence and perfusion-time interval considering temperature effect based on stress influence line, equivalent age theory, and energy method. Previous investigations on the temperature effects of an arch rib under hydration heat have focused primarily on thermal stresses. Due to the nonlinearity of the temperature field resulting from hydration heat, it becomes challenging to directly apply temperature calculation results as structural loads, which contributes to a scarcity of research on the deformation of the overall structure caused by hydration heat.

**Figure 1:**
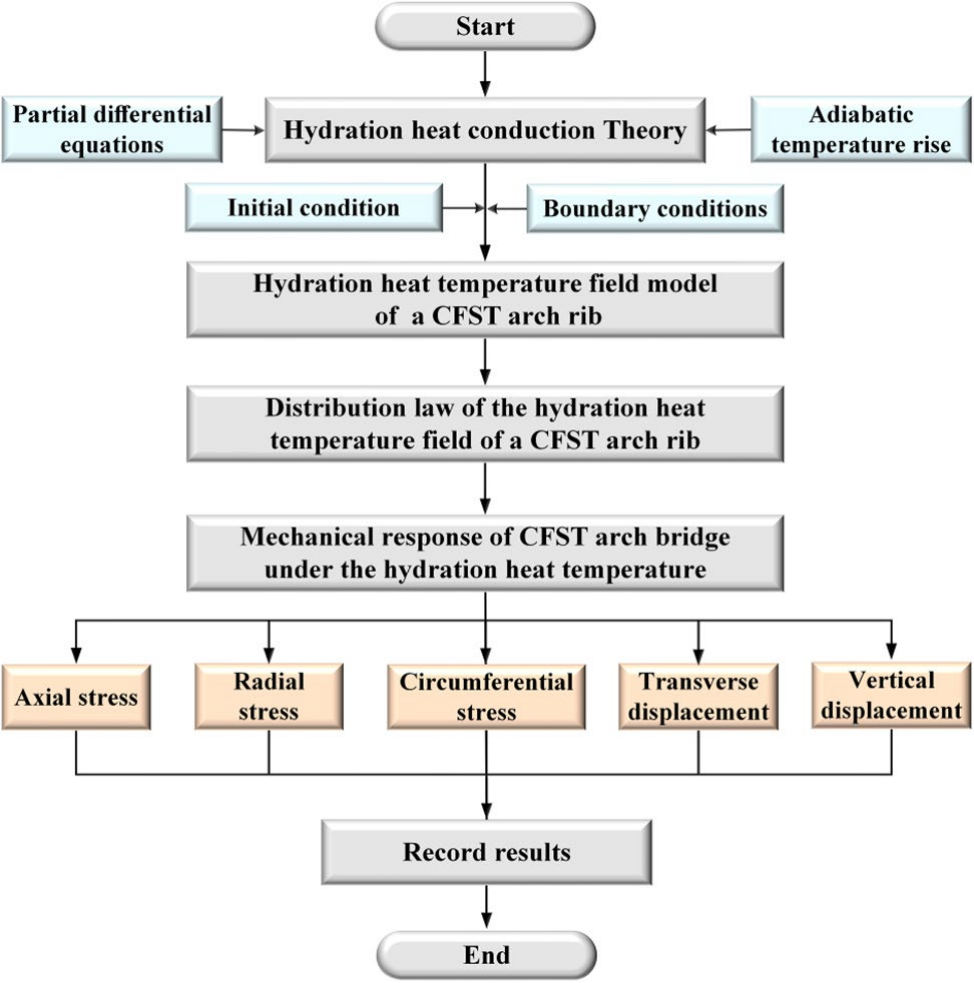
Methodology for investigating the mechanical response of a CFST arch bridge based on the hydration heat temperature effect.

In this paper, taking the Hejiang Yangtze River Highway Bridge as the research background, the mechanical response of a large-span CFST arch rib under the influence of the hydration heat temperature was investigated. First, a finite element model of the temperature field of the CFST arch rib under the effect of hydration heat was established based on hydration heat conduction theory. The distribution law of the hydration heat temperature field in the arch rib section was elucidated. Subsequently, the impact of the temperature field on the stress of the arch rib section during concrete pouring was analysed in depth. Finally, based on equivalence theory, the results of the hydration heat temperature were applied as a load in the overall structure of the arch rib, and the effect of the hydration heat temperature on the deformation of the arch rib was clarified. The methodology for investigating the mechanical response of a CFST arch bridge based on the effect of hydration heat temperature is shown in Fig. 1.

## Theory of hydration heat conduction of CFSTs

The hydration process of mass concrete is accompanied by heat release, resulting in a transient temperature field inside the CFST. Since the geometries, material parameters, and boundary conditions of the CFST have an axisymmetric distribution around the central axis^6^, the temperature field of the CFST is distributed evenly along the *ϕ*, *z* direction in the column coordinate system (*r*,*ϕ*,*z*), i.e., $\partial T/\partial \phi = \partial T/\partial z = 0$. The general partial differential equations governing the heat conduction of the CFST plane based on the energy balance principle can be presented as^27,28^

1 $$ \frac{\partial T}{{\partial t}} = \alpha \left( {\frac{{\partial^{2} T}}{{\partial r^{2} }} + \frac{1}{r}\frac{\partial T}{{\partial r}}} \right) + \frac{\partial \theta (t)}{{\partial t}} $$

where *T* refers to temperature (°C). *α* = *λ*/*ρc* denotes the thermal diffusivity of the concrete (m^2^/s). *λ*, *ρ*, and *c* represent the thermal conductivity (W/(m °C)), density (kg/m^3^) and specific heat (J/(kg °C)) of the concrete, respectively. *θ*(*t*) refers to the adiabatic temperature increase induced by the hydration heat of concrete based on the equivalent age (°C). According to the “Standard for Construction of Mass Concrete” (GB50496-2018) B.1.4, when no test data are available, *θ*(*t*) can be described as^29^

2 $$ \theta (t) = \frac{WQ}{{c\rho }}(1 - e^{mt} ) $$

where *W* denotes the dosage of cementitious material per unit volume of concrete (kg/m^3^). *Q* refers to the total hydration heat of the cementitious material (kJ/kg). *m* represents a constant.

The hydration heat conduction problem of a CFST essentially involves solving Eq. (1) under specific conditions, which include initial conditions and boundary conditions. The initial condition of the temperature field (*t* = 0) can be described as

3 $$ \left. {T(r,\phi ,z,t)} \right|_{t = 0} = T_{0} (r,\phi ,z) $$

**Figure 2:**
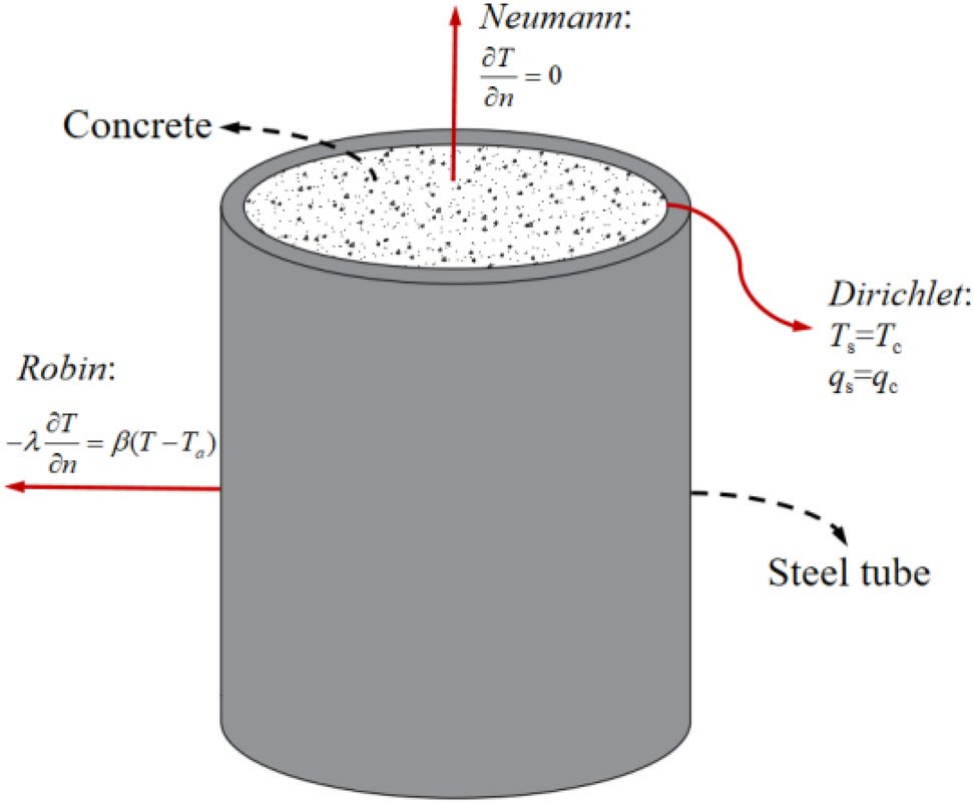
Boundary conditions for the transient temperature.

To determine the solution of the temperature field, the boundary conditions should also be satisfied. There are three types of boundary conditions for transient thermal analysis (as shown in Fig. 2), which are expressed as Eq. (4).

4 $$ \left\{ {\begin{array}{*{20}c} {Dirichlet:} & {\quad T_{s} = T_{c} ,q_{s} = q_{c} } \\ {Neumann:} & {\quad \frac{\partial T}{{\partial n}} = 0} \\ {Robin:} & {\quad - \lambda \frac{\partial T}{{\partial n}} = \beta (T - T_{a} )} \\ \end{array} } \right. $$

where *T*_s_ and *T*_c_ denote the temperature at the external surface of the concrete and the internal surface of the steel tube, respectively (°C). *q*_s_ and *q*_c_ refer to the heat flow density at the external surface of the concrete and the internal surface of the steel tube, respectively. *n* represents the normal direction outside the steel tube surface. *β* denotes the heat exchange coefficient of the steel tube. *T*_a_ refers to the ambient temperature (°C).

## Temperature field of the hydration heat of the CFST arch rib

### Project overview

**Figure 3:**
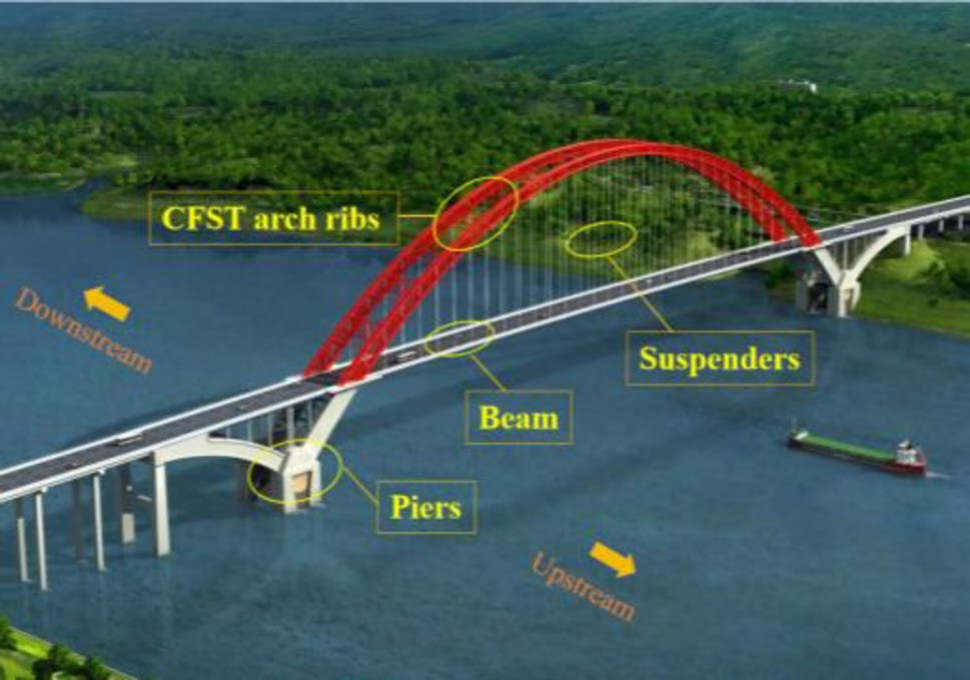
Hejiang Yangtze River Highway Bridge.

**Figure 4:**
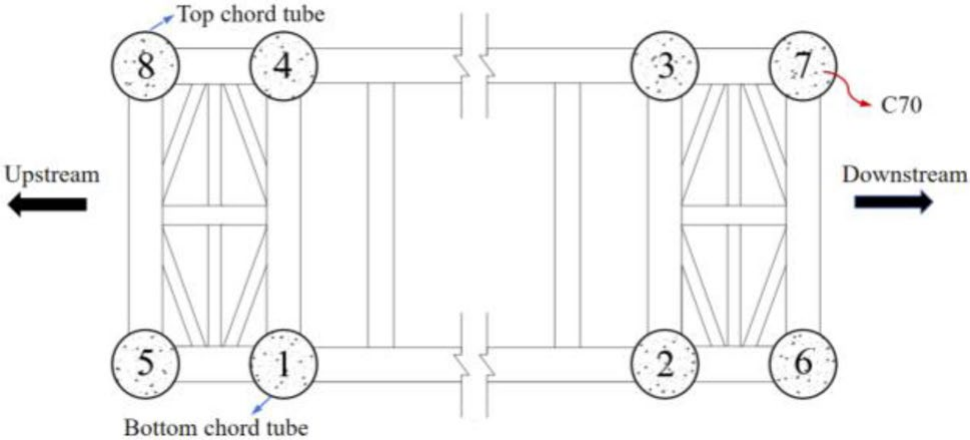
Arch rib pouring sequence.

The Hejiang Yangtze River Highway Bridge is the largest fly bird-type CFST tied arch bridge in the world. The bridge has a span arrangement of 80.5 m + 507 m + 80.5 m, with an arch rise-to-span ratio of 1/4. The arch axis adopts a catenary with an arch axis coefficient of 1.5, and the supporting project is shown in Fig. 3. The heights of the sections in the vault and arch foot are 7 m and 14 m, respectively. The diameter of the arch rib is 1.3 m, while the wall thickness of the main arch chords ranges from 22 to 30 mm. The spacing between the arch ribs is 25.3 m. A φ660 mm web steel tube and φ760 mm transverse steel tube are used between the arch ribs. The steel tubes are made of Q345 and filled with C70 high-performance concrete. The concrete is pumped in stages using the jacking-pouring method, and the perfusion sequence of the arch rib is from #1 to #8. As depicted in Fig. 4. The compressive strength of the concrete in the previous chord tube must not be less than 80% of the designed compressive strength before the concrete perfusion operation of the next arch rib.

### Finite element modelling

**Figure 5:**
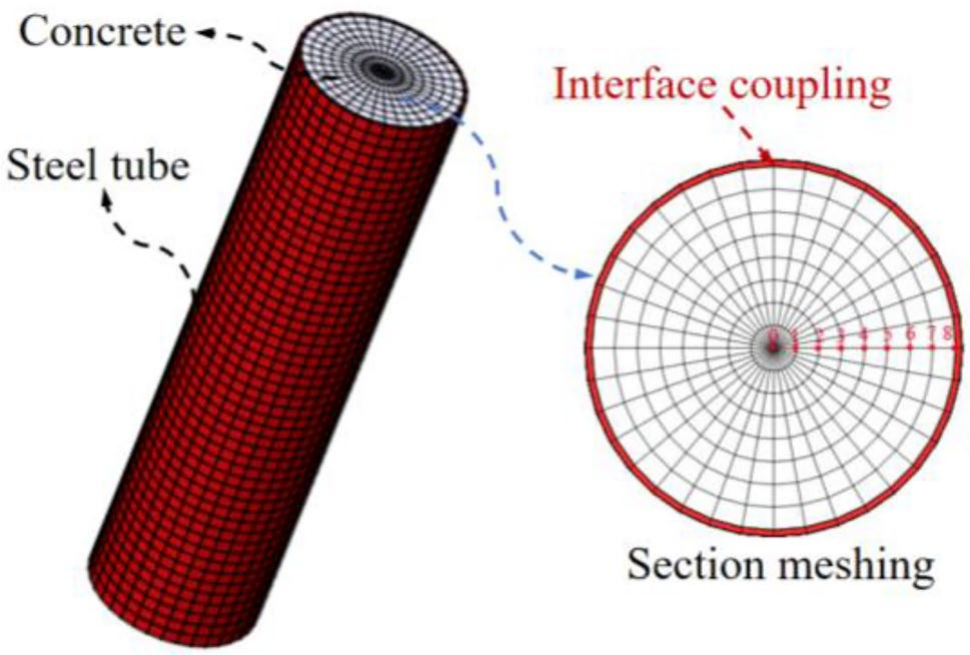
Finite element model of the hydration thermal analysis.

Midas FEA software was utilized to develop a numerical model for the transient thermal analysis of the CFST arch rib. The thermal mechanical parameters of the steel tube and concrete are presented in Table 1. The steel tube and concrete units are coupled together to ensure the continuity of heat conduction at the interface. The finite element mesh is shown in Fig. 5.

**Table 1:** Thermal and mechanical parameters.

Materials	Density (kg/m^3^)	Temperature conductivity (W·m^−1^ °C^−1^)	Heat capacity (J·kg^−1^ °C^−1^)	Thermal expansion coefficient/°C^−1^	Poisson's ratio
Concrete	2510	2.3	931	1.0 × 10^−5^	0.2
Steel	7850	48	480	1.2 × 10^−5^	0.3

### Analysis of the hydration heat temperature field

**Figure 6:**
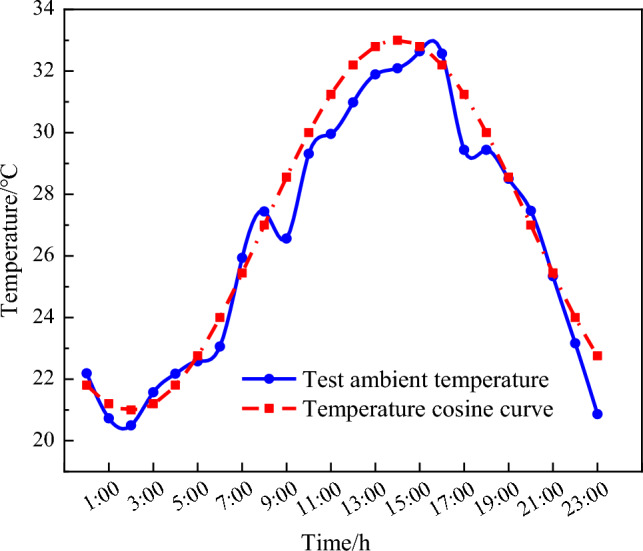
Ambient temperature curve.

**Figure 7:**
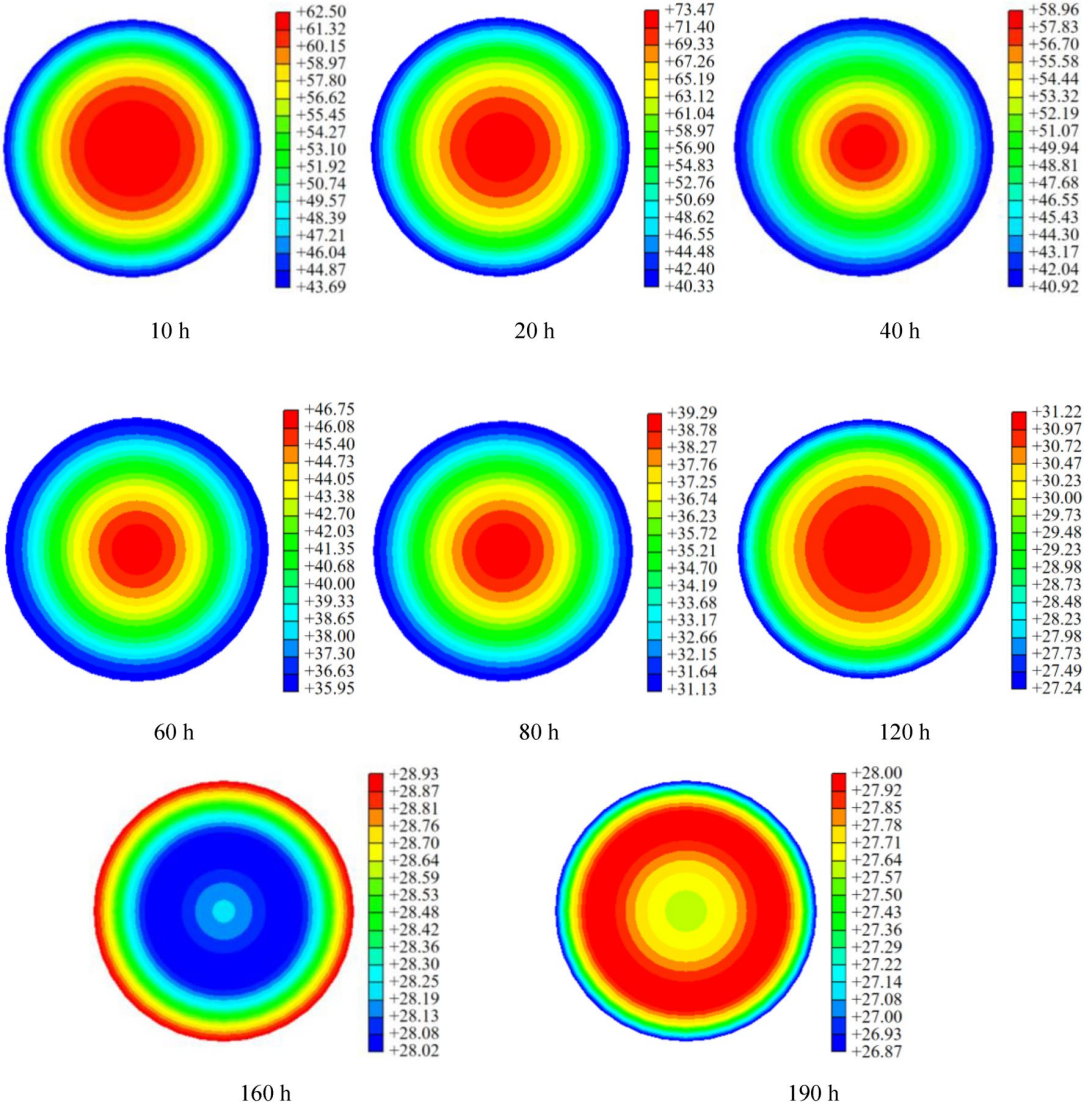
Temperature nephogram at different times.

To explore the relationship between the temperature field and time of the CFST arch rib cross section under the influence of hydration heat, the temperature field was calculated for the first 192 h after concrete perfusion by taking 5 h as a loading step. To facilitate the analysis, the ambient temperature was modelled using the cosine function, as depicted in Fig. 6. The initial temperature of the concrete was assumed to be 35 °C. The temperature nephograms at typical times are shown in Fig. 7.

Figure 7 shows that the hydration heat temperature distribution of the CFST arch rib cross section exhibits nonlinearity in both spatial and temporal dimensions. Additionally, it demonstrates axial symmetry characteristics. The main observations are summarized as follows:

1. Within 120 h after concrete perfusion, the cross section exhibits a temperature distribution pattern with higher temperatures in the centre and lower temperatures around the periphery, leading to a significant temperature gradient. Particularly at *T* = 20 h, the maximum temperature difference between the core and surface reaches 33.2 °C.
2. When T ≥ 160 h, the hydration heat of the concrete has been fully released, and the temperature variation in the section is mainly influenced by the ambient temperature, resulting in a uniform and stable temperature distribution, where the maximum temperature difference between the core and surface is less than 1 °C.

**Figure 8:**
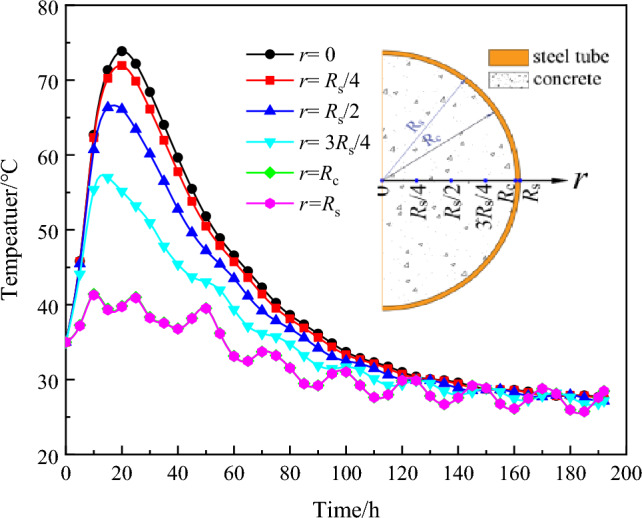
Diagram of the temperature‒time of arch rib section.

To further quantify the specific impact of hydration heat on the temperature distribution of the CFST arch rib cross section, a rectangular coordinate system is established with the centre of the section as the origin and the radius of the section as the *r*-axis. The node temperatures at *r* = 0, *R*_c_/4, 3*R*_c_/4, *R*_c_/2, *R*_c_, and *R*_s_ (*R*_c_ represents the diameter of the concrete, *R*_s_ represents the diameter of the steel tube) on the section were extracted, and the temperature‒time curves were plotted, as shown in Fig. 8.

Analysis of Fig. 8 reveals the following observations:

1. The temperature in the core concrete (*r* = 0) increases sharply at 20 h after perfusion completion, and the peak temperature reaches 73.5 °C at *T* = 20 h. Subsequently, the temperature gradually decreases, reaching 36.3 °C at T = 90 h. The concrete descending speed of the temperature between 20 and 90 h is 0.527 °C /h. At T = 190 h, the core temperature decreases to 27.6 °C, and the rate at which the temperature decreases between 90 and 190 h is 0.089 °C/h. Fluctuations in the hydration heat temperature primarily occur within 90 h after concrete perfusion.
2. Concrete located beyond *r* > 3*R*_c_/4, which is influenced by the external ambient temperature, exhibits an earlier and lower peak temperature than the core concrete. Due to the good thermal conductivity of the thin-wall steel tube, the temperature of the steel tube is basically consistent with the ambient temperature.

**Figure 9:**
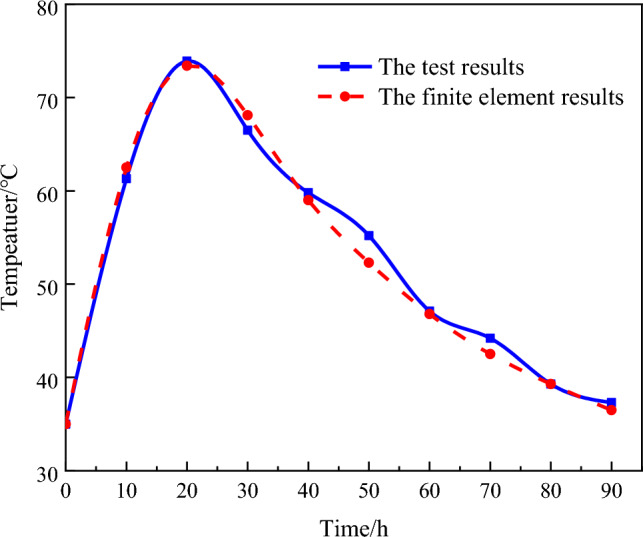
Comparison between the measured and theoretical concrete temperatures at the core point.

To verify the accuracy of the finite element simulation, the author's group carried out on-site monitoring of the core concrete temperature within 90 h after perfusion completion and compared the test results with the finite element results, as shown in Fig. 9. The numerical simulation effectively reflects the temperature field variation in the CFST arch rib during the hydration process in practical engineering.

### Radial temperature distribution of the arch rib section

**Figure 10:**
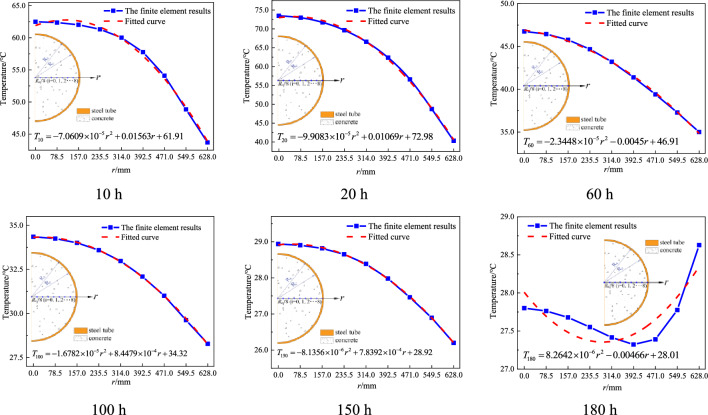
Diagram of the distribution of radial temperature at typical time.

To further clarify the radial distribution pattern of the hydration heat temperature in the cross section of the CFST arch rib, numerical fitting was performed on the diagram of the variation trends of transient temperature values for nodes located at *r* = *R*_ci_/8 (*i* = 0, 1, 2…, 8) along the radial section, as shown in Fig. 10. The fitting formulas obtained are shown in Table 2.

**Table 2:** Fitting functions.

Time/h	Fitting functions
10	$T_{10} (r) = - 7.06 \times 10^{ - 5} r^{2} + 1.56 \times 10^{ - 2} r + 61.91$
20	$T_{20} (r) = - 9.91 \times 10^{ - 5} r^{2} + 1.10 \times 10^{ - 2} r + 72.98$
60	$T_{60} (r) = - 2.34 \times 10^{ - 5} r^{2} - 4.50 \times 10^{ - 3} r + 46.91$
100	$T_{100} (r) = - 1.68 \times 10^{ - 5} r^{2} + 8.45 \times 10^{ - 4} r + 34.32$
150	$T_{150} (r) = - 8.14 \times 10^{ - 6} r^{2} + 7.84 \times 10^{ - 4} r + 28.92$
180	$T_{180} (r) = 8.26 \times 10^{ - 6} r^{2} - 4.66 \times 10^{ - 3} r + 28.01$

Based on Fig. 10, it is evident that a quadratic relationship exists between the hydration heat temperature and the radius of the cross section when *T* ≤ 150 h. The fitting curve demonstrates a high degree of agreement with the finite element results, confirming the accuracy of the fitting equation. However, for *T* ≥ 180 h, this relationship no longer holds because the temperature of the concrete is mainly affected by the ambient temperature rather than the heat of hydration at this time. Furthermore, the maximum temperature difference between the core and surface is within 0.8 °C, which can be considered a constant temperature field and is equal to the ambient temperature. In summary, the radial temperature distribution relationship of the arch ribs can be summarized as

5 $$ \left\{ {\begin{array}{*{20}l} {T(\tau ,r) = A(\tau )r^{2} + B(\tau )r + C(\tau )} & {\quad \left( {0 \le \tau \le 150} \right)} \\ {T(\tau ,r) = T_{a} (\tau ) \, } & {\quad \left( { \, \tau > 150} \right)} \\ \end{array} } \right.  $$

where $A(\tau ),B(\tau ),C(\tau )$ denote functions associated with time. $T_{a} (\tau )$ refers to a function of ambient temperature.

## Thermal stress analysis of the CFST arch rib

**Figure 11:**
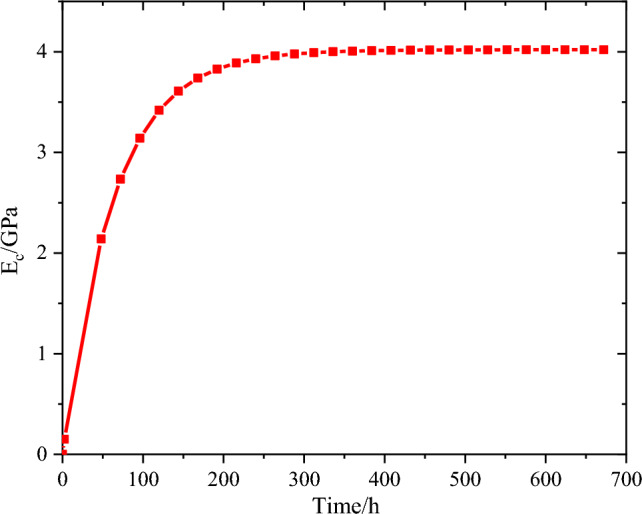
Development of the elastic modulus of the concrete.

**Figure 12:**
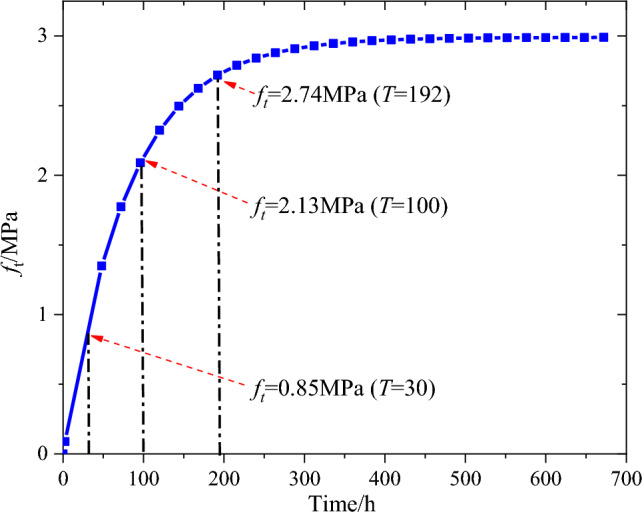
Development of the tensile strength of the concrete.

During the hydration process, concrete transforms from a fluid to a solid. As the stiffness of the concrete gradually increases, it becomes increasingly engaged in structural forces. To analyse the mechanical response of CFST arch ribs under the influence of hydration heat, it is crucial to establish a mathematical model of the mechanical properties of concrete. The elastic modulus evolution and the tensile strength evolution of the concrete are illustrated in Figs. 11 and 12^27,30^.

**Figure 13:**
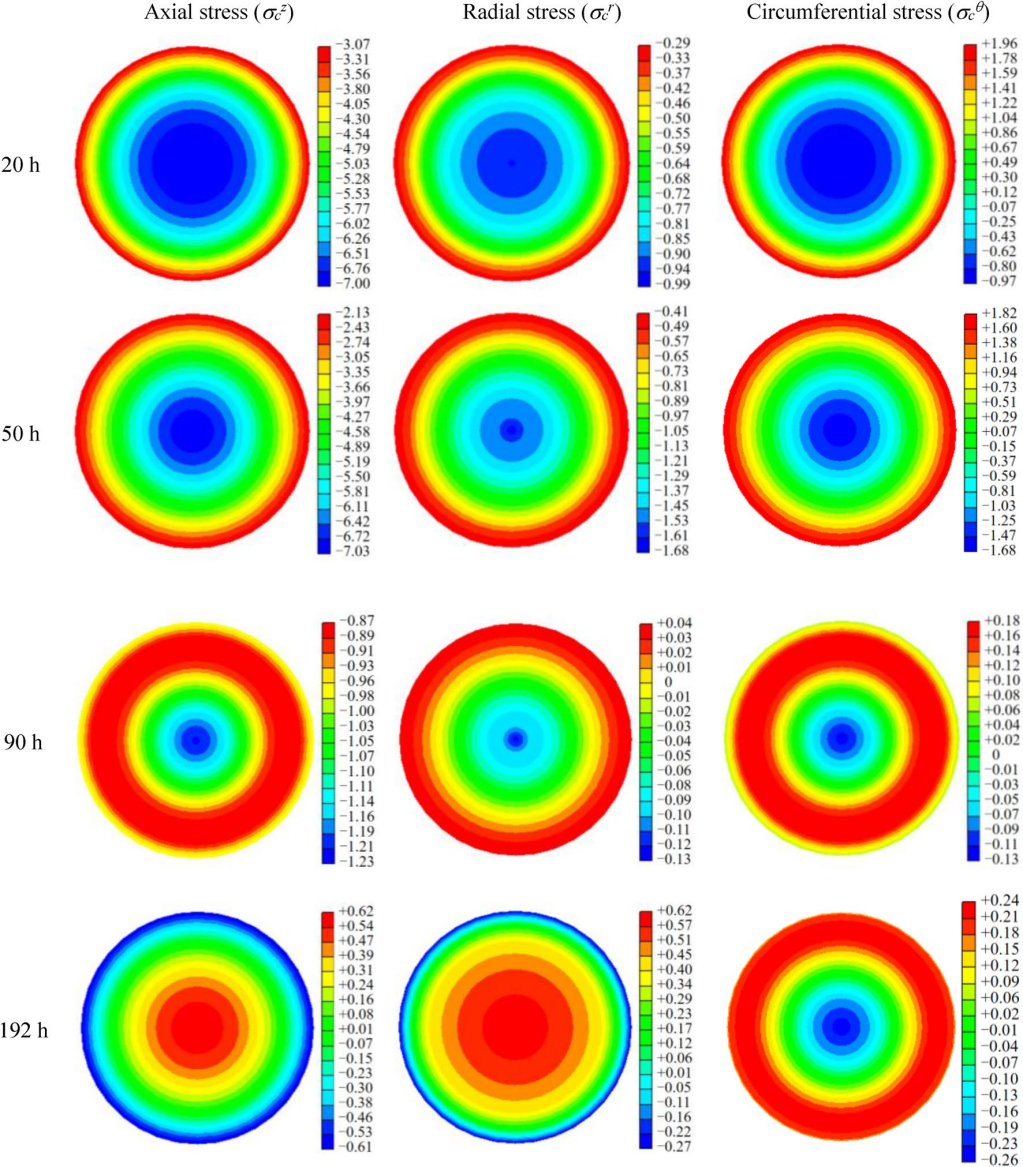
Three-dimensional stress nephogram of a concrete section at typical times (Tensile stresses were set as positive values, while compressive stresses were set as negative values).

The temperature results obtained from section “Analysis of the hydration heat temperature field” are utilized as loads for the respective nodes in structural mechanics calculations. A nephogram of the three-dimensional thermal stress (i.e., axial stress, radial stress, circumferential stress) of the concrete at typical times is shown in Fig. 13.

**Figure 14:**
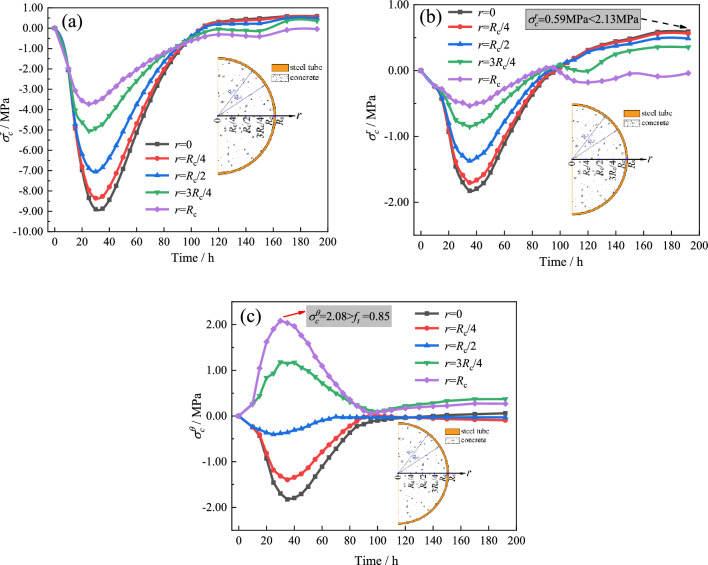
Three-dimensional thermal stress variations of the concrete: (**a**) $\sigma_{c}^{z}$, (**b**) $\sigma_{c}^{r}$, and (**c**) $\sigma_{c}^{\theta }$*.*

**Figure 15:**
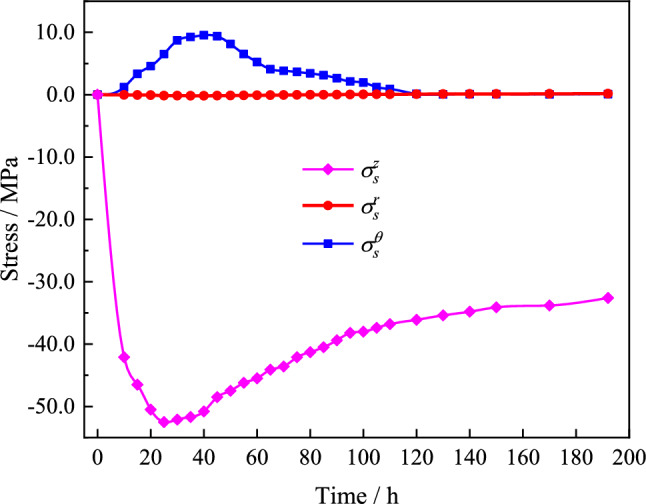
Three-dimensional thermal stress variations of the tube ($\sigma_{s}^{z}$, $\sigma_{s}^{r}$, and $\sigma_{s}^{\theta }$ denote the axial, radial, and circumferential stress, respectively).

As presented in Fig. 13, the distribution pattern of the three-dimensional thermal stress exhibits a fundamental consistency with the temperature field. To further describe the thermal stress distribution and variation characteristics of the CFST arch rib section, the three-dimensional stresses at the nodes of *r* = 0, *R*_c_/4, 3*R*_c_/4, *R*_c_/2, *R*_c_, and *R*_s_ (*R*_s_ is the diameter of the concrete, *R*_s_ is the diameter of the steel tube) on the cross section of the CFST are plotted, as shown in Figs. 14 and 15.

Figure 14 reveals that there is a large stress gradient in the cross section of the arch rib. For *T* ≤ 100 h, the axial and radial stresses of the concrete exhibit compressive stress. The maximum axial compressive stress of the core concrete is 8.9 MPa when T = 30 h, and the maximum radial compressive stress is 1.83 MPa when T = 35 h. The circumferential stress of the concrete in the region of *r* ≤ *R*_c_/2 exhibits compressive stress, but the concrete in the region of *r* > *R*_c_/2 exhibits tensile stress. Additionally, the maximum circumferential tensile stress is 2.08 MPa when T = 30 h, which exceeds the tensile strength of the concrete of 0.85 MPa at this time. This indicates the risk of concrete cracking under the influence of hydration heat.

For *T* > 100 h, the temperature of the concrete decreases to match the ambient temperature, and the temperature difference between the core and surface is negligible. The three-dimensional thermal stress in the concrete gradually approaches zero, while the radial stress in the concrete area with weaker steel tube constraints converts to tensile stress after crossing the zero-stress condition. As *r* decreases, the radial tensile stress increases, with the core concrete reaching a maximum value of 0.59 MPa, which does not exceed the tensile strength of concrete of 2.13 MPa. The radial stress in the concrete is − 0.04 MPa at *r* = *R*_c_, which is lower than the maximum bond strength between the steel tube and concrete (0.1–0.5 MPa)^31,32^, suggesting that the CFST will not experience voiding due to the effects of hydration heat.

As shown in Fig. 15, the axial stress of the steel tube increases sharply due to axial constraints at both ends in the early stage of the hydration heat. The maximum axial stress reaches 52.5 MPa when *T* = 25 h and then decreases as the temperature decreases. When *T* ≤ 120 h, the higher internal temperature of the arch rib section compared to the surface temperature causes the steel tube to be extruded by the surface concrete, resulting in circumferential tensile stress. When *T* > 120 h, the arch rib temperature matches the ambient temperature, leading to the circumferential stress in the steel tube approaching zero. Since the outer surface of the steel tube in the radial direction is unconstrained, the radial stress during the entire hydration heat process is negligible.

## Effect of the hydration heat temperature on the deformation of the CFST arch rib

The geometry of the arch rib is a crucial control factor during the construction of CFST arch bridges. A reasonable geometry can enhance the mechanical performance of the structure^33^. However, the hydration heat temperature can lead to deformation of arch ribs, thereby increasing the challenges in construction control. Therefore, it is imperative to conduct a detailed analysis of the arch rib deformation under the influence of the hydration heat temperature.

### Finite element modelling of the overall arch rib

**Figure 16:**
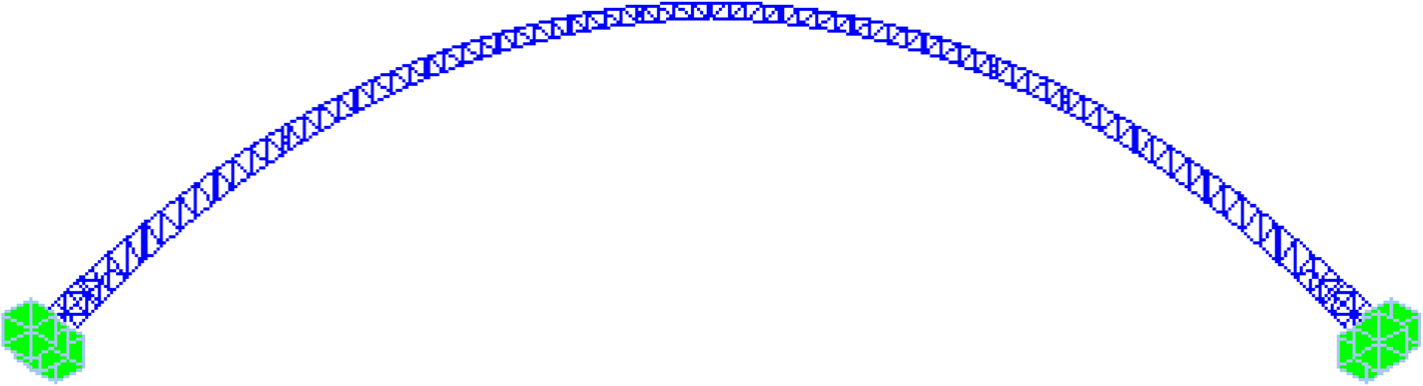
Finite element model of the overall CFST arch rib.

Midas Civil was utilized to establish the finite element model of the overall CFST arch rib. The CFST arch rib was modelled by using beam elements and the double element method. The key material parameters are presented in Table 1, and the overall structural finite element model and boundary conditions are shown in Fig. 16.

### Temperature equivalence

According to section “Analysis of the hydration heat temperature field”, the hydration heat temperature field of the arch rib cross section demonstrates significant nonlinear characteristics. However, Midas Civil only allows the application of linear temperature loads. To address this, the theory of equivalence was utilized to convert the nonlinear temperature field into a uniform temperature field and a gradient, which not only reduces the computational difficulty but also accounts for the nonlinearity of temperature.

The resultant force and moments in the cross section generated by the hydration heat temperature can be equated as

6 $$ \left\{ \begin{gathered} N^{e} = E\alpha T^{e} y \hfill \\ N = \int_{ - y/2}^{y/2} {E\alpha T(y)} dy \hfill \\ N^{e} = N \hfill \\ \end{gathered} \right. $$

7 $$ \left\{ \begin{gathered} M^{e} = \int_{ - y/2}^{y/2} {E\alpha \eta^{e} } y^{2} dy \hfill \\ M = \int_{ - y/2}^{y/2} {E\alpha T(y)} ydy \hfill \\ M^{e} = M \hfill \\ \end{gathered} \right. $$

where *N*^e^ and *M*^e^ refer to the resultant force and moments of the cross section in the equivalent temperature field, respectively. *N* and *M* denote the resultant force and moments of the cross section in the nonlinear temperature field, respectively. *T*^e^ represents the equivalent uniform temperature of the cross section. *η*^e^ refers to the equivalent temperature gradient of the cross section.* T*(*y*) denotes the function of the temperature distribution of the cross section.

According to section “Radial temperature distribution of the arch rib section”, the temperature field of the arch rib exhibits symmetry across the section. Therefore, *T*^e^ and *η*^e^ can be solved as

8 $$ T^{e} = \frac{1}{r}\int_{0}^{r} {T(r)} dr $$

9 $$ \eta^{e} = \frac{{\int_{0}^{r} {T(r)} rdr}}{{\int_{0}^{r} {r^{2} dr} }} $$

where *T*(*r*) denotes the function of the radial temperature distribution, as described in section “Radial temperature distribution of the arch rib section”.

### Transverse displacement

**Figure 17:**
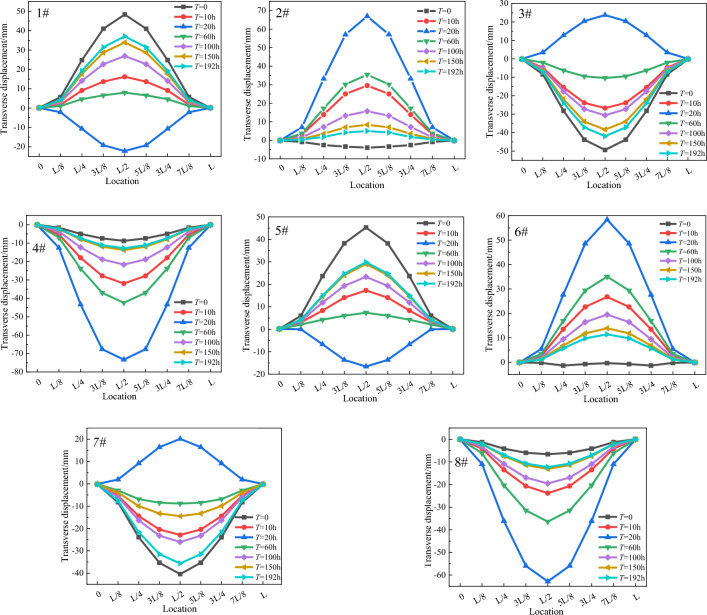
Diagram of the transverse displacement of the chord tube (L refers to the clear span of arch rib. The displacement in the upstream direction is positive, and the displacement in the downstream is negative).

To clarify the variation law of transverse displacement of each chord tube during the pouring process, the corresponding *T*^e^ and *η*^e^ were applied to the elements at different times in the overall finite element model according to the concrete perfusion construction sequence, taking into account the structural dead weight. The transverse displacement results of each tube are shown in Fig. 17.

Figure 17 shows that the transverse displacement increases progressively from the foot of the arch to the vault. No transverse displacement occurs at the foot of the arch (i.e., at 0 and L), and the maximum transverse displacement occurs at the top of the arch. The transverse displacement values of the chord tube exhibit a trend of initial increase followed by decrease with time, and the maximum transverse displacement of the chord tube occurs at *T* = 20 h (the time reaching the peak temperature). Moreover, the transverse displacement direction of odd-numbered chord tubes caused by hydration heat is opposite to that caused by gravity, whereas even-numbered chord tubes demonstrate the opposite behaviour.

**Figure 18:**
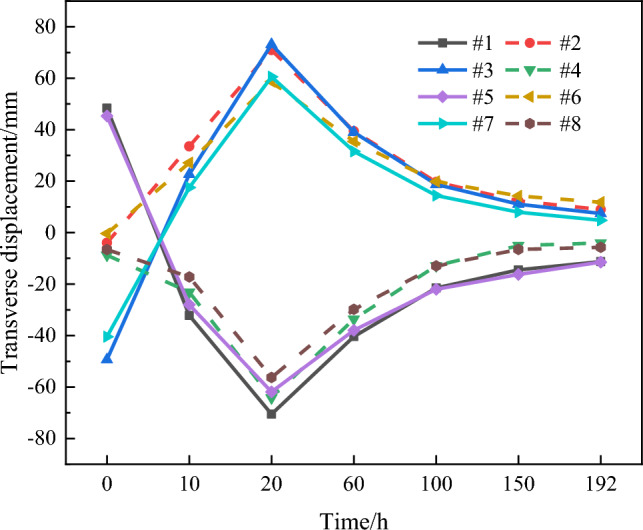
Mid-span transverse displacement of each chord tube.

The mid-span transverse displacement of each chord tube during hydration is shown in Fig. 18.

From the analysis in Fig. 18, upon completion of concrete perfusion in the odd-numbered chord tubes, the upstream and downstream sides of the arch ribs are loaded with asymmetric concrete weights, resulting in significant transverse displacement of the chord tubes. In addition, the maximum transverse displacements of chord tubes #1 and #8 are 70.6 mm and 56.3 mm, respectively, resulting in a difference of 20.3%. This is because the CFST structure is formed upon the completion of concrete perfusion in the chord tube, which increases the transverse stiffness of the single chord tube and the overall transverse stiffness of the arch rib, resulting in a decrease in the transverse displacement caused by the hydration heat temperature.

### Vertical displacement

**Figure 19:**
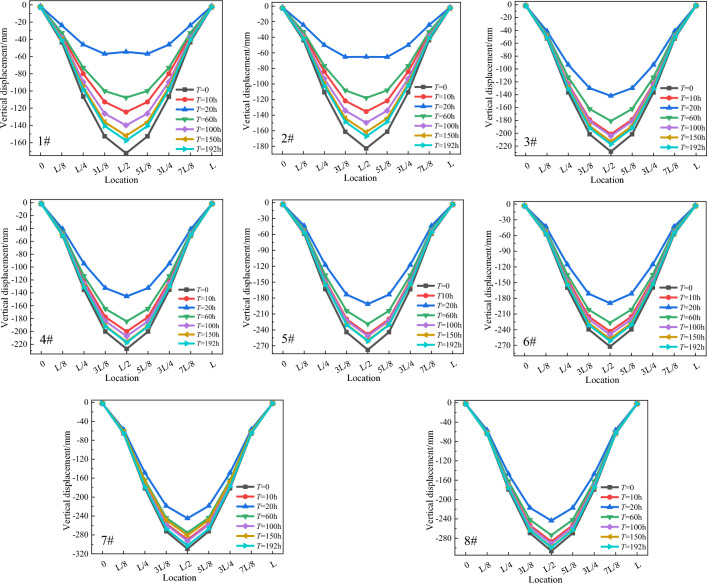
Diagram of the vertical displacement of the chord tube (L refers to the clear span of arch rib).

Similarly, we aimed to clarify the variation law of the vertical displacement of each chord tube during the perfusion process. The vertical displacement results of each chord tube are shown in Fig. 19.

As shown in Fig. 19, under the action of dead weight and hydration heat temperature, the vertical displacement increases progressively from the foot of the arch to the vault, and the vertical displacement at the arch foot approaches zero. Additionally, the vertical displacement of the chord tubes tends to first increase and then decrease, similar to the transverse displacement, with the minimum value occurring at T = 20 h. With the weakening of the hydration heat effect, the vertical displacement of the arch rib gradually translates into the balance state when the steel tube and concrete are jointly stressed.

The maximum vertical displacements of the vaults of the #1–#8 chord tubes were 117.8 mm, 117.6 mm, 86.7 mm, 81.6 mm, 86.3 mm, 83.0 mm, 64.5 mm, and 62.9 mm, respectively. The largest displacement of chord tube #1 caused by the hydration heat temperature accounts for 68.4% of that caused by the concrete self-weight. With increasing arch rib stiffness, the vertical displacement of chord tube #1 increased by 46.2% compared to that of chord tube #8.

## Conclusion

Based on hydration heat conduction theory, a finite element model of the transient temperature field of a long-span CFST arch rib under the action of hydration heat was established, and the distribution law of the hydration heat temperature of the arch rib cross section was defined. On this basis, a numerical simulation was conducted to analyse the mechanical behaviour of long-span CFST arch ribs under the influence of hydration heat. The main conclusions are summarized as follows.

1. The distribution of the hydration heat temperature in the CFST arch rib cross section exhibited nonlinear and axisymmetric characteristics in both the spatial and temporal domains. The peak temperature reached 73.5 °C, and the maximum temperature difference between the core and surface reached 33.2 °C at 20 h.
2. There was a large stress gradient in the cross section of the arch rib during the hydration process. The stress along the axial and radial directions exhibited early-age compressive stress, with maximum values of 8.9 MPa and 1.83 MPa, respectively. The radial stress of the core concrete was converted to late-age tensile stress, reaching a maximum of 0.59 MPa. The CFST remained free from voiding throughout the entire hydration process. The maximum circumferential tensile stress reached 2.08 MPa at 30 h, which indicated the risk of concrete cracking under the influence of hydration heat. It is recommended that reasonable temperature control measures be adopted during the construction process to reduce the cross-sectional temperature gradient and thus improve the performance of the CFST.
3. The displacement along the transverse and vertical directions of the chord tube tended to first increase and then decrease over time under the influence of hydration heat. The maximum transverse displacement of the chord tube reached 70.6 mm at 20 h, and the vertical displacement reached 117.8 mm, accounting for 68.4% of the displacement caused by the self-weight of the concrete.

The present study was limited to an investigation of only the temperature effect induced by the hydration heat of a CFST without considering the influence of solar radiation. However, a CFST arch bridge will inevitably be exposed to solar radiation during the construction process, which will cause the temperature field of the CFST arch rib to become more complex. In the future, further studies involving field tests or numerical simulations will be conducted considering the influence of multiple factors, which will be beneficial for the construction control of CFST arch bridges.

## Data Availability

All the data generated or analysed during this study are included in this published article.
